# Minimal change nephrotic syndrome four days after the administration of Pfizer‐BioNTech COVID‐19 vaccine—a new side effect or coincidence?

**DOI:** 10.1002/ccr3.5003

**Published:** 2021-10-23

**Authors:** Mohammed Abdulgayoom, Mhd Kutaiba Albuni, Elabbass Abdelmahmuod, Khaled Murshed, Yassir Eldeeb

**Affiliations:** ^1^ Department of Internal Medicine Hamad Medical Corporation Doha Qatar; ^2^ Division of Anatomic Pathology Hamad Medical Corporation Doha Qatar; ^3^ Department of Infectious Diseases Hamad Medical Corporation Doha Qatar

**Keywords:** COVID‐19, minimal change disease, nephrotic syndrome

## Abstract

The association between the COVID vaccine and MCD is temporal and by exclusion, and it is not fully established, but it should be considered in postvvaccine MCD.

## Graphical abstract

The association between the COVID vaccine and MCD is temporal and by exclusion, and it is not fully established, but it should be considered in post‐vaccine MCD.

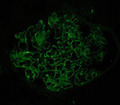

## 1 INTRODUCTION

Nephrotic syndrome is defined as the presence of massive proteinuria, hypoalbuminemia, and peripheral edema. Minimal‐change disease, which is the leading cause of nephrotic syndrome in children but less common in adults, can be primary or may occur secondary to underlying causes such as medications, infections, and various neoplasms. BNT162b2 is an mRNA‐based vaccine against coronavirus disease 2019 developed by Pfizer‐BioNTech. While common side effects of BNT162b2 have been reported in clinical trials, less common and rare side effects are increasingly recognized in parallel to the increasing number of vaccinations that are administered. We herein report a 45‐year‐old female patient with no history of renal disease who developed nephrotic syndrome due to minimal‐change disease four days after the administration of the first BNT162b2 dose. The widespread use of COVID‐19 vaccines emphasizes the importance of data collection to elucidate their potential role as triggers of glomerular diseases. Nephrotic syndrome is defined as the presence of massive proteinuria (protein excretion >3.5 g/24 h in adults), hypoalbuminemia (<3 g/dl), and peripheral edema, whereas hyperlipidemia and thrombotic diseases may also be present in some patients.^1^ Approximately 30% of individuals with nephrotic syndrome have systemic illnesses, including diabetes, amyloidosis, and systemic lupus erythematosus, whereas the remaining patients have primary diseases such as minimal‐change disease (MCD), focal segmental glomerulosclerosis, and membranous nephropathy.^2^ The main presentation of nephrotic syndrome includes edema and proteinuria, and other clinical manifestations are hypovolemia, acute kidney failure, hyperlipidemia, tendency for venous and arterial thromboses, and increased susceptibility to infections.^3^ MCD, which is the leading cause of nephrotic syndrome in children, is less common in adults, comprising approximately 10%–15% of all adult cases.^4^ MCD can be primary or secondary to underlying etiologies such as medications, infections, and various neoplasms.^5^

The Pfizer‐BioNTech coronavirus disease 2019 (COVID‐19) vaccine BNT162b2 is an mRNA‐based COVID‐19 vaccine that has been authorized for use in individuals aged twelve years and older. BNT162b2 requires two doses administered 21 days apart, although the gap between the doses has been extended in some countries.^6^ Common BNT162b2 side effects reported during clinical trials are pain and swelling at the injection site, fatigue, headache, myalgia, chills, joint pain, and fever. However, less common and rare side effects have been reported in parallel with the increasing number of individuals receiving the vaccine.^7^ We herein present the case of a 45‐year‐old female patient who developed MCD four days after the administration of the first BNT162b2 dose.

## 2 CASE PRESENTATION

A 45‐year‐old female patient with a medical history of hypothyroidism, atopic dermatitis, and a heterozygote factor V mutation and no history of kidney disease presented with a seven‐day history of swelling of lower limbs, palms, and eyelids accompanied with abdominal distension and foamy urine. She did not indicate shortness of breath, fever, flank pain, or dysuria. Four days before the onset of symptoms, the patient received the first dose of BNT162b2. Other than mild tenderness at the injection site and transient diarrhea, there were no other immediate significant adverse effects. At the time of admission, the patient was afebrile. Her blood pressure and heart rate were 126/65 mmHg and 72 beats/minute, respectively, with a normal respiratory rate, and she was maintaining oxygen saturation on room air. Physical examination revealed pitting edema in bilateral lower extremities extending to the level of thighs, swelling in both hands and around the eyelids, and abdominal ascites. Laboratory tests revealed proteinuria in the nephrotic range, hypoalbuminemia, and dyslipidemia (Table 1). Her serum creatinine level was within the normal range, and she was negative for common‐associated viral infections and autoimmune diseases. Polymerase chain reaction and serology testing for severe acute respiratory syndrome coronavirus 2 were negative. In addition to proteinuria, urinalysis revealed the presence of granular casts.

**TABLE 1:** Laboratory findings

Laboratory test	Result	Normal reference range
Serum creatinine	68 80 µmol/L	44–80 µmol/L
Urea	5.8 mmol/L	2.5–7.8 mmol/L
Cholesterol	8.3 mmol/L	≤6.2 mmol/L
LDL	6.13 mmol/L	≤4.12 mmol/L
Triglycerides	2.6 mmol/L	≤2.2 mmol/L
Serum albumin	15 g/L	35–50 g/L
HCV antibody	Non‐reactive	‐
HBV surface antigen	Non‐reactive	‐
HIV antigen/antibody	Non‐reactive	‐
Adenovirus PCR	Negative	‐
Cytomegalovirus PCR	Negative	‐
Epstein‐Barr virus PCR	Negative	
C3 level	1.24 g/L	0.9–1.8 g/L
C4 level	0.34 g/L	0.1–0.4 g/L
Anti‐cardiolipin IgM antibodies	1.90	<10
Anti‐cardiolipin IgG antibodies	2.70	<10
Anti‐β2 macroglobulin antibodies	Negative	‐
Anti‐glomerular basement membrane antibodies	1.9	<7
Anti‐neutrophil cytoplasmic antibodies	Negative	‐
Anti‐nuclear antibodies	Negative	‐
Circulating centromere antibodies	Negative	‐
Anti‐Jo−1 antibodies	Negative	‐
Anti‐Ro antibodies	Negative	‐
Total urine volume in 24 h	3000 mL	‐
24‐h creatinine in urine	9.59 mmol	7.00–14.00 mmol
24‐h protein in urine	8.73 g	0.03–0.15 g

Abbreviations

HBV, hepatitis B virus, HCV, hepatitis C virus; HIV, human immunodeficiency virus; LDL, low‐density lipoprotein; PCR, polymerase chain reaction.

Consequently, chest radiograph showed bilateral pleural effusion, transthoracic echocardiography revealed a normal ejection fraction of 62%, and ultrasonography revealed that both kidneys were normal in size, outline, and echotexture.

**FIGURE 1:**
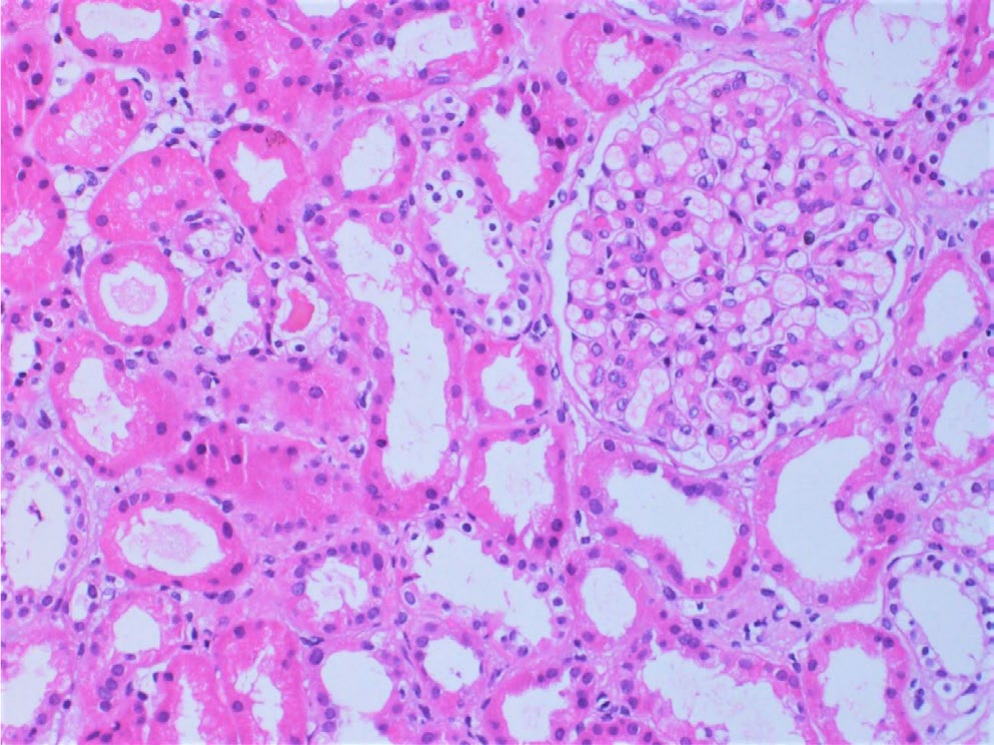
Photomicrograph of the percutaneous renal biopsy specimen stained with hematoxylin and eosin

**FIGURE 2:**
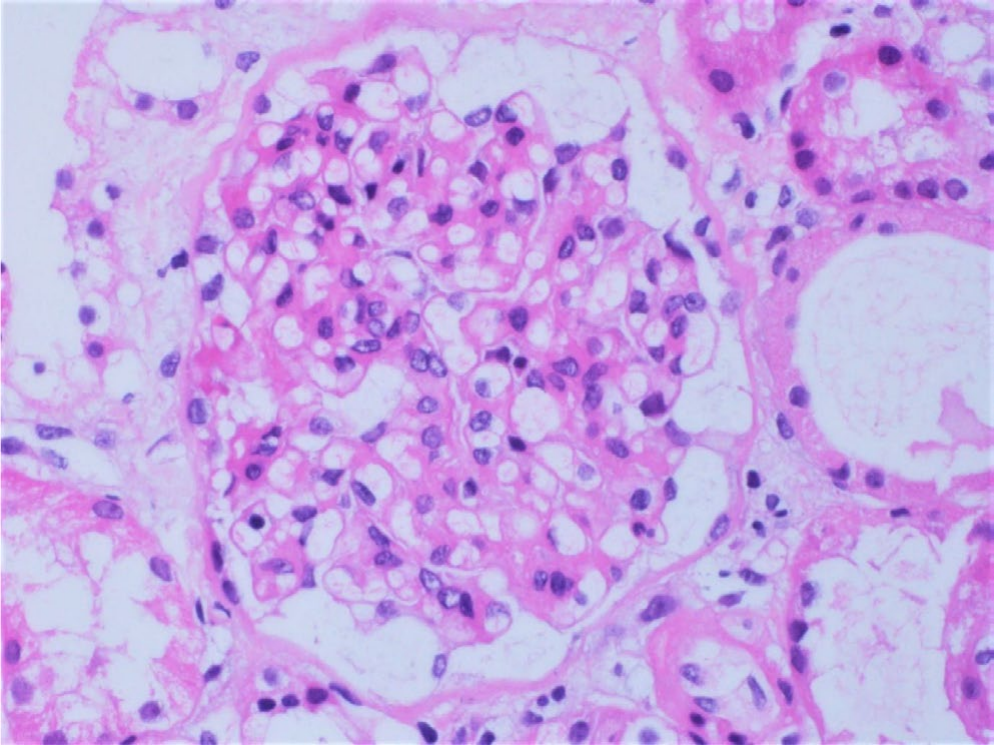
Photomicrograph of the percutaneous renal biopsy specimen stained with hematoxylin and eosin showing a glomerulus with minimal structural changes by light microscopy

**FIGURE 3:**
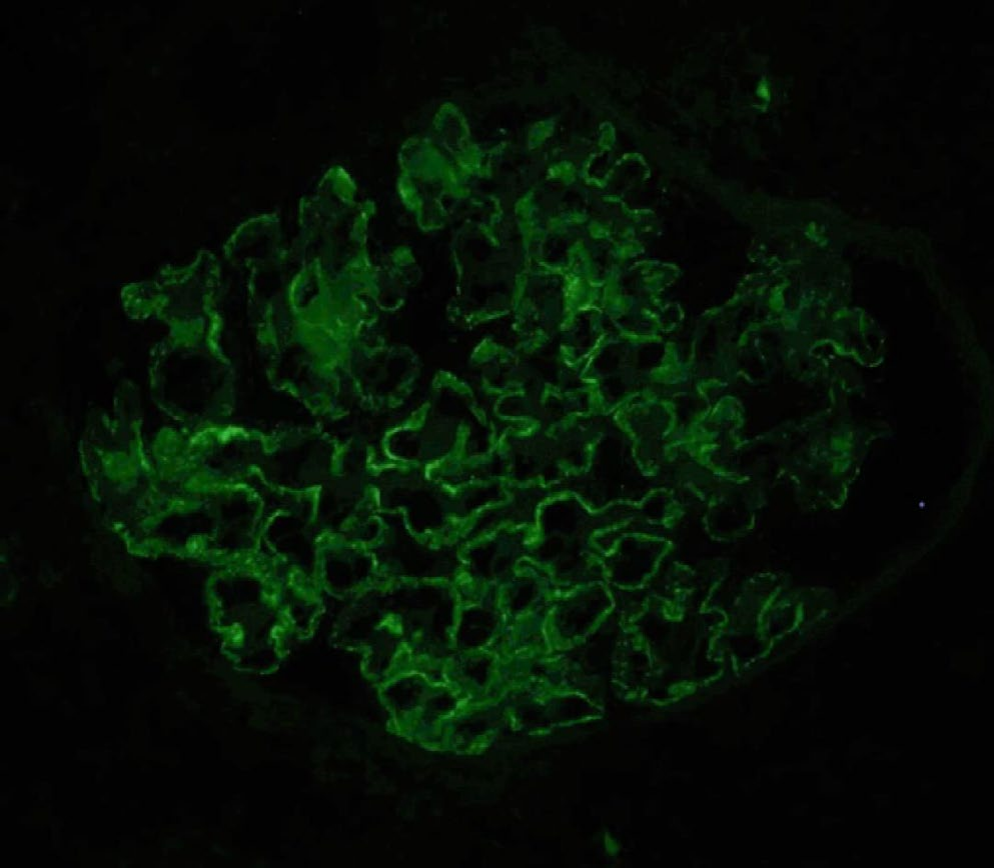
Direct immunofluorescent image of the biopsy specimen revealing the absence of glomerular deposition of immunoglobulins (IgA, IgG, and IgM)

During hospitalization, the patient was initiated on furosemide (40 mg b.i.d.) and the signs of anasarca started to improve. Her renal function tests remained stable. Histopathological examination of percutaneous renal biopsy specimens (Figures 1, 2, 3) led to the diagnosis of MCD.

The furosemide dose was later reduced to 40 mg/day p.o., and peripheral edema gradually disappeared in parallel with a loss of 8 kg. She was discharged with a treatment plan including prednisolone (60 mg/day), vitamin D, calcium, pantoprazole, and trimethoprim/sulfamethoxazole for *Pneumocystis pneumonia* prophylaxis. Follow‐up at three weeks after discharge was planned to conduct renal function tests and measurement of urine protein level.

## 3 DISCUSSION

We herein presented a middle‐aged female patient with no history of renal disease who was admitted with typical signs of nephrotic syndrome with preserved glomerular filtration rate four days after the first BNT162b2 dose. While the current case may not conclusively answer the inevitable question of a causative link between the vaccine administration and the development of MCD, several lines of evidence suggest that MCD in the current case might be related to BNT162b2. First, the onset of MCD only four days after vaccination supports a more direct link to the vaccine rather than mere coincidence. Second, the extensive workup excluded secondary causes of MCD, including medications, infections, and autoimmune diseases. Third, case reports of MCD after vaccinations against other pathogens, such as Influenza^8^ and hepatitis B virus,^9^ have been previously described. Finally, upon literature review, we found three case reports described the same presentation after the BNT162b2 vaccine, one case after the Oxford‐AstraZeneca COVID‐19 vaccine, and one case after Moderna mRNA‐1273 SARS‐Cov‐2 vaccine.

Lebedev et al.^10^ reported a 50‐year‐old man with no significant PMH who presented with nephrotic syndrome and acute kidney injury 10 days after the administration of BNT162b2 and was diagnosed with MCD based on renal biopsy findings. Additionally, D’Agati et al.(3) reported a 77‐year‐old man with a 15‐year history of type 2 diabetes without retinopathy and coronary artery disease who presented with nephrotic syndrome and acute kidney injury seven days after the administration of BNT162b2. The renal biopsy also led to the diagnosis of MCD in that patient. Furthermore, Maas et al.^11^ reported a patient in his eighties with a history of venous thromboembolism who was not on any medication. The patient presented with nephrotic syndrome seven days after the first dose of BNT162b2, and the kidney biopsy showed MCD and acute tubular injury. Leclerc et al.^12^ reported a 71‐year‐old man who presented with nephrotic syndrome 13 days after the administration of the Oxford‐AstraZeneca COVID‐19 vaccine and was diagnosed with MCD based on the kidney biopsy findings. Finally, Holzworth et al.^13^ reported a 63‐year‐old woman with a history of renal disease who developed nephrotic syndrome within a week following the first dose of the Moderna COVID‐19 vaccine; the diagnosis of MCD was based on kidney biopsy results.

The underlying pathophysiology of MCD observed in these cases may involve T cell dysfunction induced by the vaccine, similar to that observed in patients with MCD associated with other etiologies, which might explain the excellent response to steroids in some of post‐vaccine cases.^14^ Whether the second vaccine dose should be administered is another major question that requires urgent resolution. Given that the vaccine might have been a precipitating factor in the development of MCD, the second dose might trigger a more severe disease. On the contrary, despite the lack of strong evidence showing a casual link between the COVID‐19 vaccine and the development of MCD, patients with nephrotic syndrome are at high risk for the development of complications from COVID‐19 and should be fully vaccinated against COVID‐19. In this scenario, the authors believe the second dose of these vaccines would be unwise until more evidence came out.

## 4 CONCLUSION

The widespread use of COVID‐19 vaccines emphasizes the importance of data collection to elucidate their potential role in triggering glomerular diseases. Physicians, especially nephrologists, should pay attention to this possible trigger. The delay of the second vaccine dose in these circumstances may be wise until more evidence comes out.

## CONFLICTS OF INTEREST

The authors have no conflicts of interest.

## AUTHOR CONTRIBUTIONS

Mohammed Abdulgayoom involved in literature review, writing, editing, and final approving. Mhd Kutaiba Albuni involved in literature review, writing, editing, and final approving. Elabbass Abdelmahmuod involved in literature review, writing, editing, and final approving. Khaled Murshed involved in reviewing the figures. Yassir Eldeeb involved in final approving.

## ETHICAL APPROVAL

This case was approved by the Hamad Medical Corporation's Medical Research Center.

## CONSENT

Written informed consent was obtained from the patient for publication of this case report and any accompanying images.

## Data Availability

Data and materials are available on reasonable request.
